# Diffuse myocardial fibrosis and early strain abnormalities in asymptomatic type 2 diabetics without overt cardiovascular disease

**DOI:** 10.1186/1532-429X-17-S1-P261

**Published:** 2015-02-03

**Authors:** Anna Schmidt, Kelvin Chow, Madeline Arnold, Alessandro Satriano, Andrew G Howarth, Matthias G Friedrich, Richard B Thompson, James A White

**Affiliations:** Stephenson Cardiovascular Magnetic Resonance Centre, University of Calgary, Calgary, AB Canada; Alberta HEART, AI-HS Interdisciplinary Team Grant, Calgary/Edmonton, AB Canada; University of Alberta, Edmonton, AB Canada; Cardiology, Radiology, Montreal Heart Institute, Montreal, QC Canada

## Background

Type 2 diabetes has been associated with a higher prevalence of diastolic dysfunction and congestive heart failure. A hypothesized contributor is the accelerated accumulation of diffuse myocardial fibrosis. In a cohort of type 2 diabetic patients without overt cardiovascular disease, we sought to explore the capacity of quantitative T1 mapping and feature-tracking based strain analysis. These protocols were utilized to identify the presence of diffuse myocardial fibrosis and related pre-clinical systolic dysfunction. Quantitative analyses were compared to healthy age-matched controls.

## Methods

Nineteen Type 2 diabetes patients with no cardiovascular symptoms and no prior cardiac disease were recruited in addition to 13 age-matched healthy volunteers. A standardized imaging protocol was performed using a 1.5T MRI Scanner (Avanto, Siemens, Erlangen, Germany) using cine imaging, native and post-contrast T1 mapping, and late gadolinium enhancement. T1 mapping was performed using a saturation recovery single shot acquisition (SASHA) pulse sequence in basal and mid short axis views. Sequential short axis and long-axis cine images were analyzed using a prototype feature tracking strain analysis tool (cvi42, Circle Cardiovascular Inc. Calgary, Canada) to determine peak global circumferential and longitudinal systolic strain, and peak longitudinal diastolic strain rate. Segmental T1 values were determined from basal and mid slices using custom software (MATLAB), these averaged to provide a global mean T1 value for each subject. Manual exclusion of segments with visible imaging artifact was performed.

## Results

Among diabetic subjects, the mean age was 57±9 years, 7 (39%) being female, and Hb_A1c_ range 7.5-9.9%. The mean age of healthy volunteers was 52±12 years, 2 (15%) being female. The left ventricular ejection fraction was normal among all diabetic and healthy controls (57.9±2.6% versus 57.8±2.9%, respectively, p=NS). Strain analysis was successful in all subjects and showed trends towards reduction among diabetics in peak longitudinal systolic strain (-15.2±1.5 vs -16.1±1.3%, p=0.08). Late gadolinium enhancement imaging was visually normal in all subjects with no evidence of focal injury. There was a significant increase in native myocardial T1 values in diabetics compared to age-matched controls (1176±32 ms vs 1149±20ms, p=0.0096) and decrease in post-contrast T1 values (578±64 ms vs 628±41 ms, p=0.019), both of which are consistent with the presence of diffuse myocardial fibrosis.

## Conclusions

Quantitative T1 mapping in asymptomatic type 2 diabetic patients showed changes consistent with increased myocardial fibrosis, as compared to controls. A trend towards reduced systolic strain indices was also seen, despite preservation of global ejection fraction. These imaging markers show promise for the detection of pre-clinical cardiomyopathy in patients with type 2 diabetes.

## Funding

Ms. Schmidt receives MD-PhD support from Alberta Innovates-Health Solutions. Funding was additionally provided, in part, by an AI-HS Interdisciplinary Team Grant via Alberta HEART. Dr White is supported a Heart and Stroke Foundation of Alberta Early Investigator Award.

